# Establishment and verification of a nomogram that predicts the risk for coronary slow flow

**DOI:** 10.3389/fendo.2024.1337284

**Published:** 2024-03-04

**Authors:** Jiang Yu, Yangshan Ran, Dan Yi, Chengyu Yang, Xiang Zhou, Sibin Wang, Hao Li, Wensi Yu, Zhijun Sun, Zhengbo Zhang, Muyang Yan

**Affiliations:** ^1^ Department of Hyperbaric Oxygen, The First Medical Centre of Chinese People’s Liberation Army (PLA) General Hospital, Beijing, China; ^2^ Graduate School, Chinese People’s Liberation Army Medical School, Beijing, China; ^3^ Department of Internal Medicine, Xuanhan Chinese Medicine Hospital, Dazhou, Sichuan, China; ^4^ West China School of Public Health and West China Fourth Hospital, Sichuan University, Chengdu, Sichuan, China; ^5^ Department of General Medicine, Zhige Township Hospital, Meishan, Sichuan, China; ^6^ Graduate School, Guangxi University of Chinese Medicine, Nanning, Guangxi, China; ^7^ Department of Cardiology, The First Medical Centre of Chinese People’s Liberation Army General Hospital, Beijing, China; ^8^ Center for Artificial Intelligence, Medical Innovation and Research Department, Chinese People’s Liberation Army General Hospital, Beijing, China

**Keywords:** coronary slow flow, nomogram, coronary angiography, prediction, diagnosis

## Abstract

**Background:**

Coronary slow flow (CSF) has gained significance as a chronic coronary artery disease, but few studies have integrated both biological and anatomical factors for CSF assessment. This study aimed to develop and validate a simple-to-use nomogram for predicting CSF risk by combining biological and anatomical factors.

**Methods:**

In this retrospective case-control study, 1042 patients (614 CSF cases and 428 controls) were randomly assigned to the development and validation cohorts at a 7:3 ratio. Potential predictive factors were identified using least absolute shrinkage and selection operator regression and subsequently utilized in multivariate logistic regression to construct the nomogram. Validation of the nomogram was assessed by discrimination and calibration.

**Results:**

N-terminal pro brain natriuretic peptide, high density lipoprotein cholesterol, hemoglobin, left anterior descending artery diameter, left circumflex artery diameter, and right coronary artery diameter were independent predictors of CSF. The model displayed high discrimination in the development and validation cohorts (C-index 0.771, 95% CI: 0.737-0.805 and 0.805, 95% CI: 0.757-0.853, respectively). The calibration curves for both cohorts showed close alignment between predicted and actual risk estimates, demonstrating improved model calibration. Decision curve analysis suggested high clinical utility for the predictive nomogram.

**Conclusion:**

The constructed nomogram accurately and individually predicts the risk of CSF for patients with suspected CSF and may be considered for use in clinical care.

## Introduction

After decades of exploration and research, clinical physicians have gradually come to accept that coronary slow flow (CSF) is an independent clinical entity characterized by unique epidemiological characteristics, clinical manifestations, and prognosis (1). It distinguishes itself from slow blood flow or no-reflow resulting from percutaneous coronary intervention (PCI) (2). However, the pathophysiology of CSF remains unclear and may be linked to factors such as gender, lifestyle, impaired carbohydrate and lipid metabolism, alterations in cardiac and coronary anatomical structures, and changes in blood composition (3–5). Some studies have indicated a relatively low incidence of CSF in coronary angiography, with patients frequently presenting risk factors such as smoking, obesity, and metabolic syndrome1. The main clinical manifestations are recurrent unstable angina, chest tightness, chest pain, and other chest discomfort symptoms (6, 7). These symptoms often recur days or months after discharge, significantly impacting the life quality of patients. In addition, patients with CSF are often susceptible to arrhythmia, myocardial infarction, and even sudden death (8–10). Unfortunately, specific treatments for CSF are currently lacking (1).

Easy-to-use, well-validated tools for clinical diagnosis of CSF are important in clinical care, in particular for treatment decisions in primary prevention. Regrettably, to date, there has been limited progress in enhancing the utility of diagnostic tools by incorporating cardiac influencing factors. Nomograms, which are simple and accurate visualization tools, have found extensive application in predicting the risk of coronary artery diseases. Nevertheless, there have been few studies integrating cardiac risk factors into nomograms for assessing the risk of CSF. In this study, we sought to develop and validate a nomogram including cardiac factors for predicting the risk of CSF.

## Materials and methods

### Patient population

**Figure 1:**
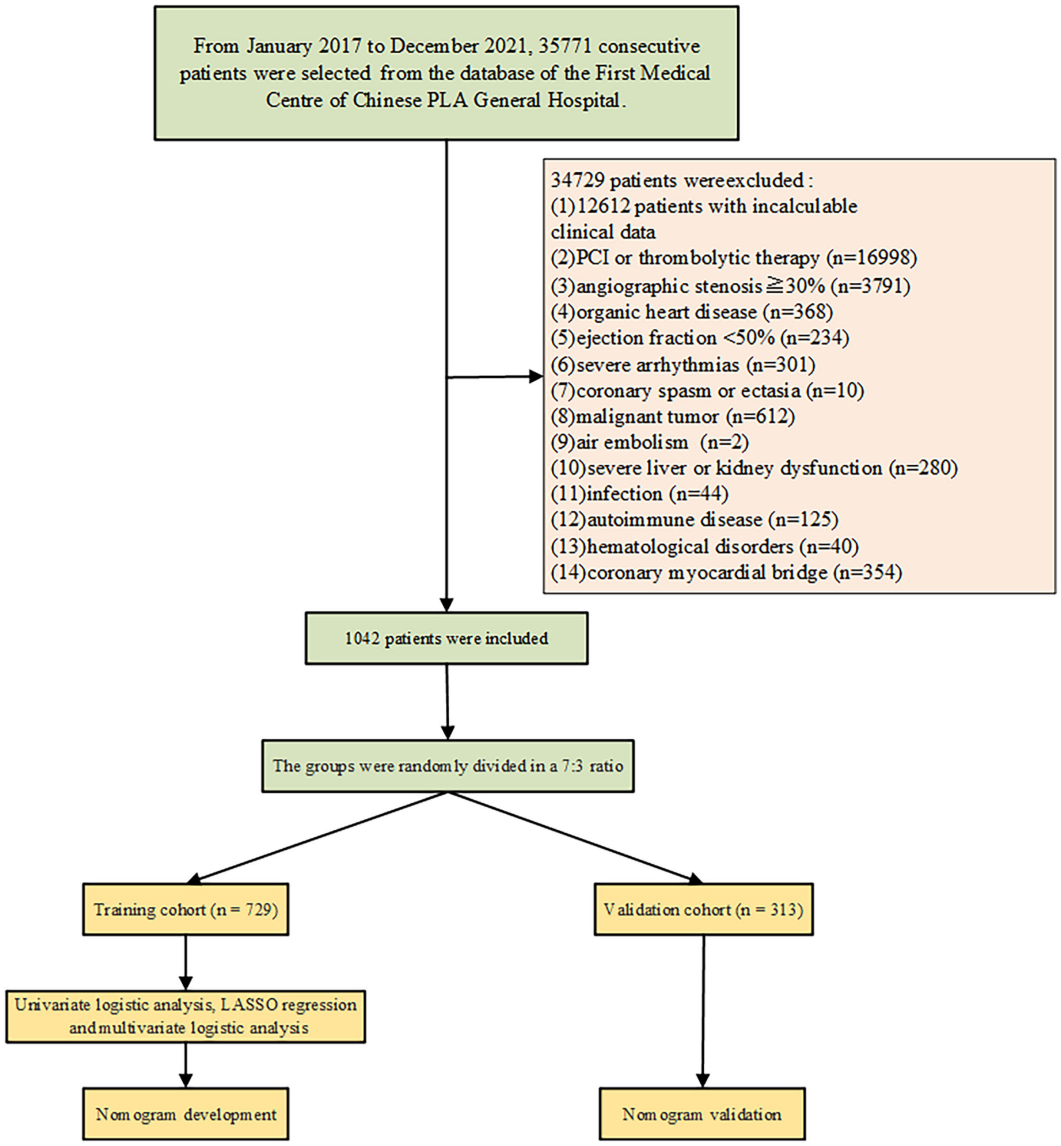
Selection diagram of all patients enrolled in the study.

The retrospective cohort study was performed by using collected data from the First Medical Centre of Chinese PLA General Hospital. The inclusion criteria: the study included patients with normal or near-normal coronary vessels (angiographic stenosis<30%) during the coronary angiography. The exclusion criteria: Patients with incomplete clinical or angiographic data, percutaneous coronary intervention (PCI) and Stenting, no-reflow and slow flow after PCI, thrombolytic therapy, organic heart disease (e.g. cardiomyopathy, severe valvular heart disease, congenital heart disease), left ventricular systolic dysfunction (ejection fraction <50%), significant arrhythmias (e.g. second degree or higher atrioventricular block, pacemaker implantation, and cardioverter defibrillator implantation), coronary spasm, coronary ectasia, malignant tumors, air embolism during coronary angiography, severe liver or kidney dysfunction, infection, autoimmune diseases, hematological disorders, coronary myocardial bridge were excluded. A total of 35,771 consecutive patients were enrolled between January 1, 2017, and December 31, 2021 (**Figure 1**). 1042 patients were included according to the inclusion criteria. Among them, 614 patients were identified as CSF and 428 patients as non-coronary slow flow (NCSF).

### Ethical considerations

The study was approved by the medical ethics committee of the Chinese PLA General Hospital and abided by the ethical principles of the Declaration of Helsinki. Written informed consent was obtained from all participants.

### Coronary angiography

The corrected thrombolysis in myocardial infarction frame count (CTFC), a quantitative and objective measure, was employed to assess coronary blood flow. Coronary angiography (CAG) was performed by the standard Judkins technique and through the radial or femoral approach in all patients. The first frame was determined when the injected contrast medium completely filled the entrance of the coronary artery. The final frames were considered to be that the contrast agent reached the distal landmark branch of every coronary artery, including the left anterior descending artery (LAD), left circumflex artery (LCX), and right coronary artery (RCA). The distal LAD bifurcation (“whale’s tail”, “figure eight” branch), the distal bifurcation of the obtuse marginal branch of LCX, and the first branch of the posterolateral branch of RCA were considered standard signs. The CTFC values were recorded and then counted at the speed of 15 frames per second. Therefore, the CTFC attained was multiplied by two. The obtained values for the LAD were divided by 1.7 to obtain a modified CTFC, because the LAD was longer than other coronary arteries. Patients were recognized as having CSF, if the CTFC exceeded 2 standard deviations from the normal range for at least one among three major coronary arteries.

### Data extraction

Demographic variables (age and sex), and traditional coronary artery disease (CAD) risk factors (hypertension, hyperlipidemia, diabetes, smoking history, and alcohol consumption) were collected. Body mass index (BMI), systolic blood pressure (SBP), diastolic blood pressure (DBP), heart rate, height, weight, and body surface area (BSA) were recorded for all participants.

Information on the following laboratory test results was collected: total cholesterol (TC), triglyceride (TG), high density lipoprotein cholesterol (HDL-C), LDL-C: low density lipoprotein cholesterol, hemoglobin (HBG), white blood cell count (WBC), neutrophil ratio, lymphocyte ratio, platelet count, alanine aminotransferase (ALT), aspartate aminotransferase (AST), troponin T (cTnT), glucose, urea, creatinine, uric acid, creatine kinase (CK), creatine kinase isoenzyme (CK-MB), N-terminal pro brain natriuretic peptide (NT-proBNP), neutrophil-lymphocyte ratio (NLR), systemic immune-inflammation index (SII). The SII was calculated using the following formula: (neutrophil count × platelet count)/lymphocyte count. NLR was calculated from the neutrophil count/lymphocyte count.

The data of echocardiography were recorded, including left ventricular end-diastolic diameter (LVEDD), left ventricular end-systolic diameter (LVESD), ejection fraction (EF), interventricular septal end-diastolic thickness (IVSd), left ventricular posterior wall end-diastolic thickness (LVPWd), left atrial anteroposterior diameter (LA-ap), mitral valve E peak velocity, mitral valve A peak velocity, mitral valve E/A ratio, aortic valve peak systolic velocity (AV-v), left ventricular mass (LVM). The following formula was used to calculate left ventricular mass: Left ventricular mass = 0.8×1.04× [(IVSd + LVEDD + LVPWd)^3^-(LVEDD)^3^] +0.6.

### Coronary diameter

Data collection training was carried out for cardiovascular intervention doctors. Upon successfully passing the training, doctors proceeded to collect data based on specific diagnostic criteria. The angiography image was analyzed offline at the imaging disk workstation, employing measurement tools for assessing coronary artery diameter. Proximal diameters of the three major coronary arteries were assessed using a computerized quantitative coronary angiography analysis system (Philips Medical System, Philips, the Netherlands).

### Statistical analysis

Data was collected using EpiData software version 3.1, and then the statistical analyses were performed using SPSS statistical software version 26.0 and R software version 4.3.1. Descriptive statistics were presented as mean, standard deviation, frequency and percentage, or median and interquartile ranges (P25, P75). The Kolmogorov-Smirnov test was performed to assess whether the quantitative variables were normally distributed. The independent sample test and Mann–Whitney U test were used to compare the continuous variables. Chi-square test and Fisher’s exact test were used to compare the categorical variables. The subjects were randomly divided into the development cohort and the validation cohort at a ratio of 7:3. The influencing factors related to CSF were screened by using the least absolute shrinkage and selection operator (LASSO) regression. Multivariate logistic regression analysis was performed to analyze the influencing factors of CSF. A nomogram was developed by using weighted estimators corresponding to each covariate derived from fitted Logistic regression coefficients and estimates of variance. Validation of the nomogram was assessed by discrimination and calibration. Harrell’s C statistic was calculated by 1,000-fold bootstrap resampling iterations to an initial fitted Logistic model in the development cohort. These development estimates were then applied to yield a Harrell’s C statistic in the validation cohort. The calibration curve was used to assess the discriminatory ability and calibration of the model. The clinical utility of the model was assessed using decision curve analysis (DCA). A probability value <0.05 was considered statistically significant.

## Results

### Characteristics of the development cohort and the validation cohort

Among the 35771 patients enrolled in the First Medical Centre of Chinese PLA General Hospital, 1042 patients met the above inclusion criteria and were randomly divided into the development cohort (n=729) and the validation cohort (n=313) at a ratio of 7:3. There were no statistically significant differences in clinical characteristics or laboratory test results between the groups (**Table 1**). This demonstrated that the separation of our dataset is reasonable and comparable.

**Table 1:** Baseline characteristics of training and validation cohorts.

Variable	development cohort (n=729)	validation cohort (n=313)	*p*-value
Male sex, n (%)	395 (54.2)	176 (56.2)	0.543
Hypertension, n (%)	360 (49.4)	144 (45.9)	0.346
Diabetes, n (%)	108 (14.8)	42 (13.4)	0.502
Hyperlipidemia, n (%)	132 (18.1)	60 (19.2)	0.685
Smoking history, n (%)	262 (35.9)	121 (38.7)	0.404
Alcohol consumption, n (%)	257 (35.3)	115 (36.7)	0.646
Age (year)	57 ± 10	56 ± 10	0.416
Heart rate (bpm)	76 ± 12	77 ± 12	0.600
SBP (mmHg)	131 ± 17	130 ± 17	0.196
DBP (mmHg)	76 ± 11	76 ± 11	0.787
Height (cm)	166.7 ± 8.1	167.0 ± 8.7	0.530
Weight (kg)	73.1 ± 13.5	73.4 ± 13.7	0.672
BMI (kg/m^2^)	26.2 ± 3.7	26.2 ± 3.6	0.921
LVEDD (mm)	44.65 ± 3.89	44.38 ± 3.72	0.307
LVESD (mm)	29.86 ± 3.17	29.47 ± 2.91	0.066
EF (%)	61.59 ± 4.83	62.10 ± 4.72	0.119
IVSd (mm)	10.66 ± 1.18	10.61 ± 1.17	0.544
LA-ap (mm)	34.32 ± 3.95	34.31 ± 4.01	0.957
Mitral valve E peak velocity (m/s)	0.65 ± 0.16	0.66 ± 0.16	0.607
Mitral valve A peak velocity (m/s)	0.77 ± 0.17	0.76 ± 0.18	0.467
AV-v (m/s)	1.31 ± 0.24	1.29 ± 0.25	0.231
Mitral valve E/A ratio	0.88 ± 0.28	0.90 ± 0.30	0.212
LVM (g)	236.3 ± 48.5	232.4 ± 50.3	0.239
Urea (mmol/l)	5.31 ± 1.41	5.26 ± 1.38	0.620
Creatinine (µmol/l)	73.0 ± 14.8	72.8 ± 15.5	0.896
Uric acid (µmol/l)	331.9 ± 88.9	335.8 ± 91.6	0.519
Potassium (mmol/l)	3.73 ± 0.33	3.72 ± 0.35	0.942
TC (mmol/l)	4.10 ± 0.93	4.18 ± 1.00	0.081
HDL-C (mmol/l)	1.15 ± 0.31	1.15 ± 0.31	0.905
LDL-C (mmol/l)	2.51 ± 0.82	2.61 ± 0.8	0.058
HBG (g/l)	139.2 ± 15.1	139.3 ± 15.1	0.957
WBC (10^9/L)	6.32 ± 1.60	6.24 ± 1.62	0.445
Neutrophil ratio	0.58 ± 0.90	0.57 ± 0.09	0.071
Lymphocyte ratio	0.32 ± 0.09	0.33 ± 0.08	0.067
Platelet count (10^9/L)	221.5 ± 55.8	219.0 ± 52.7	0.498
Diameters of coronary arteries			
LCA (mm)	4.44 ± 0.85	4.44 ± 0.90	0.968
LAD (mm)	3.47 ± 0.67	3.45 ± 0.66	0.646
LCX (mm)	3.11 ± 0.66	3.07 ± 0.75	0.459
RAD (mm)	3.42 ± 0.72	3.38 ± 0.72	0.317
CTFC			
LAD (frames)	36 ± 16	36 ± 17	0.870
LCX (frames)	29 ± 14	30 ± 15	0.239
RCA (frames)	30 ± 15	31 ± 17	0.283
ALT (U/l, Median [IQR])	18.1 (13.5-26.2)	19.4 (13.0-28.5)	0.974
AST (U/l, Median [IQR])	16.9 (14.2-20.5)	16.6 (14.1-22.0)	0.427
cTnT (ng/ml, Median [IQR])	0.006 (0.004-0.008)	0.006 (0.004-0.008)	0.930
Glucose (mmol/l, Median [IQR])	5.31 (4.82-6.02)	5.35 (4.78-5.93)	0.436
CK (U/L, Median [IQR])	76.2 (58.9-105.8)	76.2 (55.8-102.6)	0.409
Myoglobin (U/L, Median [IQR])	24.0 (21.0-30.3)	23.4 (21.0-30.7)	0.642
CK-MB (ng/ml, Median [IQR])	1.18 (0.86-1.62)	1.16 (0.82-1.63)	0.493
NT-proBNP (pg/ml, Median [IQR])	46.4 (24.4-92.7)	46.9 (24.3-90.1)	0.989
TG (mmol/l, Median [IQR])	1.40 (1.05-1.95)	1.41 (1.06-1.95)	0.740
NLR (Median [IQR])	1.84 (1.40-2.46)	1.78 (1.31-2.29)	0.089
SII (Median [IQR])	387 (292-530)	382 (270-517)	0.130

### Baseline patient characteristics in development cohort

Baseline clinical characteristics of the nomogram derivation cohort are presented (**Table 2**). This study found that there was significantly more prevalence of men, smoking history, and alcohol consumption in the CSF group compared to the NCSF group. The study group had a significantly higher height, weight, BMI, DBP, LVEDD, LVESD, IVSd, LA-ap, LVM, creatinine, uric acid, HBG, WBC, neutrophil ratio, AST, ALT, CK, TG, NLR than the control group, while age, mitral valve E peak velocity, mitral valve A peak velocity platelet count, lymphocyte ratio, and HDL-C were significantly lower in the study group. Patients with CSF had larger coronary arteries and higher CTFC in each of the major coronary arteries compared to the control group. The clinical characteristics including the presence of hypertension, hyperlipidemia, and diabetes were not statistically significant different between the CSF and NCSF groups. Similarly, the parameters incorporating heart rate, SBP, DBP, EF, AV-v, mitral valve E/A ratio, urea, potassium, TC, cTnT, Glucose, creatine kinase isoenzyme, myoglobin, CK-MB, NT-proBNP, and SII did not differ significantly between groups (**Table 2**).

**Table 2:** Baseline characteristics of the CSF group and NCSF group.

Variable	NCSF (n=294)	CSF(n=435)	*p*-value
Male sex, n (%)	118 (40.1)	277 (63.7)	<0.001
Hypertension, n (%)	163 (54.5)	213 (49.0)	0.086
Diabetes, n (%)	52 (17.7)	80 (18.4)	0.809
Hyperlipidemia, n (%)	45 (15.3)	63 (14.5)	0.759
Smoking history, n (%)	81 (27.6)	181 (41.6)	<0.001
Alcohol consumption, n (%)	76 (25.9)	181 (41.6)	<0.001
Age (year)	58 ± 11	56 ± 10	0.041
Heart rate (bpm)	77 ± 11	76 ± 12	0.115
SBP (mmHg)	131 ± 18	131 ± 16	0.266
DBP (mmHg)	75 ± 11	77 ± 11	0.022
Height (cm)	164.4 ± 7.6	168.2 ± 8.1	<0.001
Weight (kg)	69.5 ± 12.9	75.4 ± 13.5	<0.001
BMI (kg/m^2^)	25.63 ± 3.80	26.54 ± 3.64	0.001
LVEDD (mm)	44.09 ± 3.81	45.03 ± 3.91	0.001
LVESD (mm)	29.50 ± 3.08	30.10 ± 3.21	0.011
EF (%)	61.67 ± 4.62	61.54 ± 4.97	0.724
IVSd (mm)	10.49 ± 1.17	10.77 ± 1.17	0.001
LA-ap (mm)	33.84 ± 3.81	34.64 ± 4.01	0.007
Mitral valve E peak velocity (m/s)	0.67 ± 0.16	0.64 ± 0.16	0.004
Mitral valve A peak velocity (m/s)	0.79 ± 0.16	0.76 ± 0.17	0.009
AV-v (m/s)	1.32 ± 0.24	1.30 ± 0.24	0.254
Mitral valve E/A ratio	0.88 ± 0.26	0.88 ± 0.29	0.953
LVM (g)	227.3 ± 46.6	242.3 ± 48.9	<0.001
Urea (mmol/l)	5.21 ± 1.44	5.38 ± 1.39	0.115
Creatinine (µmol/l)	69.6 ± 13.7	75.2 ± 15.1	<0.001
Uric acid (µmol/l)	316.9 ± 84.1	342.0 ± 90.7	<0.001
Potassium (mmol/l)	3.71 ± 0.32	3.74 ± 0.34	0.246
TC (mmol/l)	4.09 ± 0.93	4.05 ± 0.93	0.587
HDL-C (mmol/l)	1.21 ± 0.33	1.11 ± 0.29	<0.001
LDL-C (mmol/l)	2.53 ± 0.84	2.49 ± 0.80	0.522
HBG (g/l)	134.9 ± 13.9	142.1 ± 15.1	<0.001
WBC (10^9/L)	6.16 ± 1.61	6.44 ± 1.59	0.023
Neutrophil ratio	0.58 ± 0.09	0.59 ± 0.09	0.020
Lymphocyte ratio	0.33 ± 0.08	0.31 ± 0.08	0.033
Platelet count (10^9/L)	227.2 ± 56.1	217.6 ± 54.3	0.022
Diameters of coronary arteries			
LCA (mm)	4.15 ± 0.80	4.64 ± 0.82	<0.001
LAD (mm)	3.21 ± 0.62	3.65 ± 0.65	<0.001
LCX (mm)	2.85 ± 0.56	3.28 ± 0.66	<0.001
RAD (mm)	3.16 ± 0.66	3.60 ± 0.71	<0.001
CTFC			
LAD (frames)	22 ± 4	46 ± 15	<0.001
LCX (frames)	19 ± 5	35 ± 15	<0.001
RCA (frames)	19 ± 5	37 ± 17	<0.001
ALT (U/l, Median [IQR])	16.9 (12.1-23.9)	19.2 (14.3-28.1)	0.001
AST (U/l, Median [IQR])	16.2 (14.0-20.1)	17.2 (14.7-20.9)	0.037
cTnT (ng/ml, Median [IQR])	0.006 (0.004-0.008)	0.006 (0.004-0.008)	0.222
Glucose (mmol/l, Median [IQR])	5.34 (4.77-6.04)	5.34 (4.86-5.98)	0.889
CK (U/L, Median [IQR])	72.2 (56.3-96.0)	79.6 (60.4-110.5)	0.060
Myoglobin (U/L, Median [IQR])	22.8 (21.0-28.9)	24.0 (21.0-31.3)	0.182
CK-MB (ng/ml, Median [IQR])	1.14 (0.83-1.57)	1.23 (0.88-1.69)	0.098
NT-proBNP (pg/ml, Median [IQR])	50.20 (26.0-92.0)	43.3 (24.1-93.4)	0.267
TG (mmol/l, Median [IQR])	1.32 (1.00-1.75)	1.50 (1.07-2.08)	0.002
NLR (Median [IQR])	1.78 (1.33-2.37)	1.87 (1.45-2.50)	0.033
SII (Median [IQR])	385 (285-525)	389 (295-541)	0.518

### LASSO regression and multivariate logistic analyses

**Figure 2:**
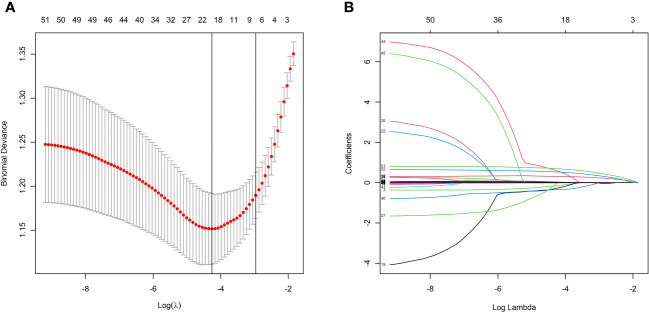
LASSO regression model screening predictors of CSF. **(A)** LASSO regression model cross-validation plot. Draw a vertical line at the optimum with the minimum criterion and 1se of the minimum criterion. 19 variables were selected when the most available parameter value λ = 0.0141. **(B)** Coefficient profile plot of predictors was performed against the log (λ) sequence.

LASSO regression analysis showed that hypertension, heart rate, height, weight, IVSd, mitral valve E peak velocity, cTnT, serum creatinine, blood urea, NT-proBNP, TC, HDL, hemoglobin, white blood cell count, neutrophil ratio, platelet count, left anterior descending artery diameter, left circumflex artery diameter, and right coronary artery were the more important predictors in patients with CSF (**Figure 2**). These 19 variables were evaluated by using multivariate logistic regression analysis to identify independent factors significantly associated with CSF. The results showed that NT-proBNP, HDL, left anterior descending artery diameter, left circumflex artery diameter, and right coronary artery were independent predictors of CSF (**Table 3**). Among these, HDL was a protective factor, while the remaining variables were considered risk factors (**Table 3**).

**Table 3:** Multivariate logistic regression analysis for risk factors of CSF.

Variable	β	SE	Wald	*P*	OR	95%CI
HDL	-0.722	0.286	6.393	0.011	0.486	0.277-0.850
HBG	0.026	0.006	17.475	<0.001	1.026	1.014-1.039
LAD diameter	0.411	0.180	5.177	0.023	1.508	1.059-2.147
LCX diameter	0.785	0.179	19.212	<0.001	2.192	1.543-3.114
RCA diameter	0.627	0.140	19.946	<0.001	1.871	1.421-2.463
NT-proBNP	0.001	0.001	3.917	0.048	1.001	1.000-1.003

### Development of the nomogram

**Figure 3:**
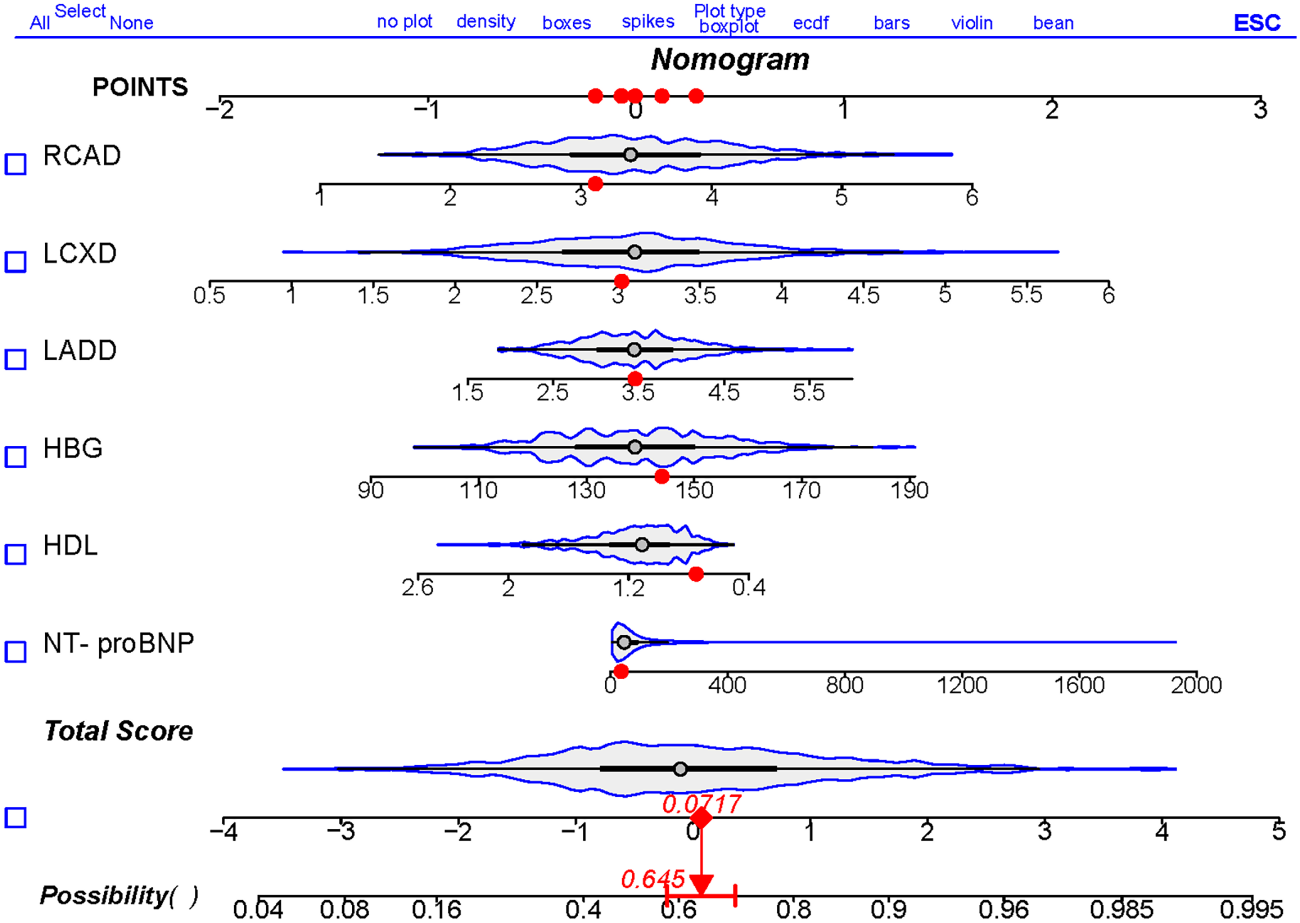
Nomogram to predict the risk of CSF. Draw a line perpendicular from the corresponding axis of each risk factor until it reaches the top line labeled “POINTS”. Sum up the number of points for all risk factors then draw a line descending from the axis labeled “Total Score” until it intercepts diagnostic probabilities. NT-proBNP, N-terminal pro brain natriuretic peptide; HDL, high density lipoprotein cholesterol; HBG, hemoglobin; LADD, left anterior descending artery diameter; LCXD, left circumflex artery diameter; RCAD, right coronary artery diameter.

Based on the above analysis, a nomogram was created to predict the probability of CSF using NT-proBNP, HDL, left anterior descending artery diameter, left circumflex artery diameter, and right coronary artery as predictors (**Figure 3**). Each patient was assigned points based on the presence of these factors. The sum of these points (“total points”) is converted to a probability of CSF.

### Validation of the nomogram

**Figure 4:**
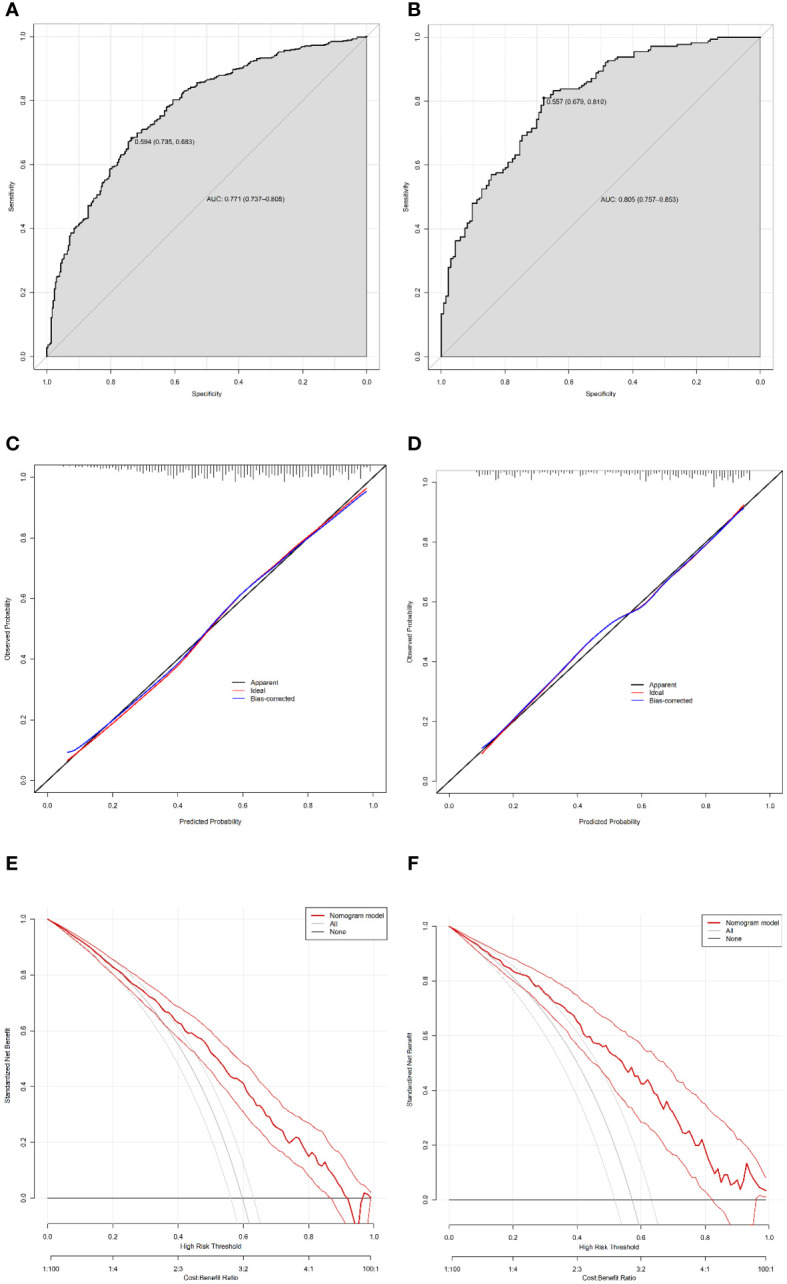
Validation of the nomogram. Harrell’s C-index of the predictive accuracy of the nomogram for CSF in the development **(A)** and independent validation cohorts **(B)**. Calibration curve of the nomogram on the data of the development cohort **(C)** and validation cohort **(D)**. Decision curve analysis of the nomogram in the development **(E)** and validation **(F)** cohorts.

Harrell’s C-index was performed to analyze the discriminatory capacity of the nomogram model. Harrell’s C-index for the development cohort was 0.771 (95% CI: 0.737-0.805, **Figure 4A**). Applying the development cohort estimates to the validation cohort yielded a similar Harrell’s C-index of 0.805 (95% CI: 0.757-0.853, **Figure 4B**). This indicates that there was an outstanding discriminative ability in the nomogram model. Bootstrap self-sampling method with B = 1000 repetitions and the calibration curves were performed to assess the valid predictive accuracy of the nomogram for the development and validation cohorts. The patterns of both plots showed good agreement between the predicted and observed probabilities of CSF, indicating suitable model calibration (**Figures 4C, D**). Decision curve analysis (DCA) was used to measure the clinical utility of the nomogram model. DCA presented that when the threshold probability was approximately 23–91%, the model had a good overall net benefit in the development cohort (**Figure 4E**). Additionally, the nomogram had an overall net benefit within a wider threshold probability in the validation cohort (**Figure 4F**). Those results suggested that the nomogram holds potential clinical validity.

## Discussion

CSF was first discovered by Tambe et al. in 1972 in patients undergoing coronary angiography due to chest pain (3). These patients exhibited normal coronary artery structure, but experienced slow blood flow velocity. Although CSF is currently considered an independent chronic coronary syndrome, the pathogenetic mechanisms of CSF still remain uncertain. Importantly, in previous studies, several hypotheses have been proposed including endothelial dysfunction, subclinical coronary atherosclerosis, microcirculation disorders, chronic inflammation, and oxidative stress (1, 4, 11–13). In addition, recent studies have also shown that CSF could be associated with coronary artery anatomical factors and gene polymorphisms (14–16).

In the comparison of clinical data between the two groups, this study found that there was significantly more prevalence of man, smoking history, and alcohol consumption and a significantly higher height, weight, BMI, DBP, and TG in the CSF group, which is in good agreement with earlier reports (6, 17, 18). Numerous studies have suggested that CSF was associated with male gender, smoking, and metabolic risk factors (higher TC and LDL-C levels, impaired glucose metabolism, lower HDL-C levels, and higher BMI) (18–20). Cardiovascular diseases are more prevalent in male patients, but women develop these diseases about a decade later than men. This phenomenon may be related to the effect of estrogen on improving vascular endothelial function and reducing LDL-C (21, 22). However, most men have histories of smoking and drinking. The nicotine and tar in tobacco can lead to endothelial damage, vasospasm, and decreased blood oxygen-carrying capacity (21). Additionally, traditional risk factors of cardiovascular diseases, including lipid and glucose metabolism abnormalities and hypertension, are also involved in endothelial dysfunction, vascular wall thickening, and atherosclerosis formation. These factors can contribute to myocardial ischemia and hypoxia due to weight gain, increased BMI, left ventricular wall thickening, diastolic function deterioration, and myocardial microcirculation disturbances (23).

However, in the multivariate analysis, this study did not show that male gender, hyperlipidemia, smoking history, drinking history, weight, BMI, and DBP were independent risk factors of CSF, which was related to the fact that males accounted for the main part of the included population and were greatly affected by their diet and lifestyle. The prospective studies were required to verify the above influencing factors.

In this study, the patients with CSF had significantly higher values of NT-proBNP and the NT-proBNP were identified as independent predictors of CSF. While the role of NT-proBNP in the pathogenic mechanisms of CSF still remains completely unclear, the previous study demonstrated that elevated NT-proBNP has been highly associated with patients with CSF (24). It has been shown that the NT-proBNP is secreted primarily from cardiomyocytes in response to ischemia or increased wall stress. Elevated levels of serum NT-proBNP are widely used as significant indicators to predict clinical diagnosis, mortality, and hospitalization in patients with cardiac dysfunction, coronary hypoperfusion, and cardiovascular disease (25, 26). Candemir et al. suggested that the levels of NT-proBNP are higher in patients with CSF, particularly those with scar tissue (24). The secretion of BNP from fibroblasts of scar tissue induces matrix metalloproteinases, leading to fibrosis (27). Elevated NT-proBNP in CSF may be associated with impaired coronary blood flow, which can induce myocardial ischemia and subsequent BNP secretion. Moreover, the increased wall stress in increased LV filling pressures is another potential cause of elevated NT-proBNP levels. This study also revealed that patients with CSF had significantly higher LVEDD and LVESD, further supporting the role of increased wall stress in NT-proBNP elevation. Additionally, histological and pathological changes in the coronary arteries may also induce the ventricular myocardium to release BNP.

This study observed that the diameter of the coronary arteries was larger in patients with CSF than in those with NCSF and was associated with high CSF risk, which is consistent with previous studies (14, 15). According to Bernoulli’s equation and Poiseuille’s law, the flow velocity of Newtonian fluids in circular-sectioned pipes decreases when the pipes abruptly enlarge28 (28). Thus, changes in cardiovascular diameter or cross-sectional area may play an important role in the pathophysiology of CSF. The enlarged coronary artery may be associated with early atherosclerosis, chronic inflammation, and endothelial dysfunction (14). In terms of myocardial pathology, Mangieri et al. have conducted a myocardial biopsy of the left ventricle in patients with CSF, revealing small coronary vessels with features such as medial hypertrophy, fibromuscular hyperplasia, myofibril disarray, mitochondrial abnormalities, and decreased intracellular glycogen (29). In addition, the structural abnormalities in coronary arteries mainly leaded to luminal narrowing and functional obstruction, including thickening of the arteries, fibromuscular hyperplasia, endothelial swelling, and increased resting microvascular resistance in coronary vessels <400µm (30–32). These factors are impairing myocardial blood supply, resulting in unstable angina pectoris in normal or near-normal coronary arteries. Nie et al. showed that the tortuosity index and the number of distal branches of the three major coronary arteries were significantly increased, significantly correlated with the mean CTFC, and independent predictors of CSF (14). Furthermore, using multidetector CT coronary angiography, Kantarci et al. found a significant correlation between CSF and a small angle of origin of the main coronary arteries from the aorta (15). Finally, Yigit et al. reported that carotid and coronary artery diameters were significantly elevated in patients with CSF, suggesting carotid artery dilatation may be used as an early indicator for CSF (33).

Patients with CSF often present with recurrent unstable angina pectoris, arrhythmias, and cardiovascular and cerebrovascular events, and they generally have a poor prognosis (6, 34). Therefore, early identification of patients with high risk of CSF is crucial for defining preventive treatment strategies and improving the quality of life and survival prognosis for these individuals. This study included NT-proBNP, HDL, hemoglobin, left anterior descending coronary artery diameter, left circumflex artery diameter, and right coronary artery diameter as prediction factors to develop a nomogram model. The verification results showed that it had high distinguishing ability, agreement, and clinical application value.

This study complies with several limitations. The study was conducted as a single-center retrospective case-control study. This design may introduce selection bias, as all subjects were referred by physicians. Consequently, the findings may not be fully generalizable to broader populations. Additionally, this study had a small sample size due to the low incidence of CSF in coronary angiography. The limited number of these study individuals could impact the robustness of the conclusions drawn. The limited number of clinical indicators collected may introduce bias in the study of risk factors of CSF. Moreover, as this study was the first to establish a nomogram for estimating the probability of CSF, there were few similar studies to refer to during its development. Finally, multicenter external validation of the nomogram model was not performed, which limited the extrapolation of the model. Therefore, prospective, multicenter, and large-sample studies are needed to yield more robust results in the future. In light of these limitations, the findings of this study should be interpreted with caution.

In conclusion. This study developed and validated a nomogram that incorporates six clinical characteristics, including NT-proBNP, HDL, left anterior descending artery diameter, left circumflex artery diameter, and right coronary artery. Those variables are both straightforward to obtain and usually collected in CSF risk assessment. The nomogram serves as a practical tool, offering clinicians a user-friendly method to directly assess the risk of CSF in their patients.

## Data availability statement

The raw data supporting the conclusions of this article will be made available by the authors, without undue reservation.

## Ethics statement

The studies involving humans were approved by the medical ethics committee of the Chinese PLA General Hospital. The studies were conducted in accordance with the local legislation and institutional requirements. Written informed consent for participation in this study was provided by the participants’ legal guardians/next of kin.

## Author contributions

JY: Conceptualization, Data curation, Formal analysis, Funding acquisition, Investigation, Methodology, Project administration, Resources, Software, Supervision, Validation, Visualization, Writing – original draft, Writing – review & editing. YR: Data curation, Investigation, Methodology, Project administration, Writing – original draft. DY: Conceptualization, Data curation, Formal analysis, Methodology, Resources, Software, Supervision, Validation, Writing – original draft. CY: Conceptualization, Funding acquisition, Investigation, Methodology, Project administration, Software, Supervision, Writing – original draft. XZ: Data curation, Investigation, Methodology, Project administration, Resources, Visualization, Writing – original draft. SW: Data curation, Formal analysis, Investigation, Project administration, Software, Supervision, Writing – original draft. HL: Conceptualization, Formal analysis, Investigation, Methodology, Resources, Software, Validation, Writing – original draft. WY: Conceptualization, Formal analysis, Funding acquisition, Methodology, Project administration, Resources, Validation, Writing – original draft. ZS: Investigation, Methodology, Supervision, Validation, Writing – review & editing. ZZ: Supervision, Validation, Visualization, Writing – review & editing. MY: Writing – review & editing.
